# Protocol for a feasibility and pilot study of the implementation and impact of specialist multi-agency teams supporting children and young people at risk of, or experiencing, violence or criminal exploitation outside the home

**DOI:** 10.1186/s40814-025-01736-z

**Published:** 2025-11-25

**Authors:** Cheryl McQuire, Harry Sumnall, Jane Harris, Frank de Vocht, Nadia Butler, Zara Quigg

**Affiliations:** 1 School of Public and Allied Health/Public Health Institute, Faculty of Health, Liverpool John Moores University, 3rd Tithebarn Building, Liverpool, L3 2ER UK; 2 School of Psychology/Public Health Institute, Faculty of Health, Liverpool John Moores University, Liverpool, UK; 3 Centre for Public Health, Population Health Sciences, University of Bristol, Bristol, UK; 4 NIHR Applied Research Collaboration West (NIHR ARC West), Bristol, UK

**Keywords:** Feasibility, Pilot, Children, Youth, Violence, Exploitation, Multi-agency teams

## Abstract

**Background:**

Across the United Kingdom (UK), there are increasing calls for the implementation of multi-agency approaches to addressing violence or criminal exploitation outside the home (i.e. extra-familial harm) that address the needs of the child/young person (and their families) and the neighbourhood context in which harms occur. However, to date, there is very little evidence on what an effective multi-agency approach to supporting children and young people, and their families, looks like, or the services they should provide. This article presents the protocol for a feasibility and pilot study of a specialist multi-agency team embedded in neighbourhoods to support children and young people, and their families, who are at risk of, or experiencing, violence or criminal exploitation outside the home.

**Methods:**

A mixed-methods feasibility and pilot study will examine implementation across five UK sites. Pre- and post-outcome measures will be collected from ~1000 children/young people receiving targeted support (~200 per site). Interviews will be undertaken with children and young people, parents/carers, and stakeholders to examine views and experiences of programme implementation and outcomes/impacts, and as relevant evaluation design and outcome measurements. The extent to which findings from the feasibility and pilot study support progression to a full impact study will be reviewed. If a randomised controlled trial (RCT) is not feasible, we will explore a quasi-experimental (natural experiment) evaluation design, using the ‘Target Trial Framework’ to make explicit where a future evaluation will align with, and where it deviates, from the ideal target trial (RCT).

**Discussion:**

This study will provide an important and timely contribution to the emerging, but limited, evidence base surrounding the implementation of place-based multi-agency interventions to support children, young people, and their families at risk of extra-familial harm. This work has direct implications for informing UK policy and practice in the wake of the Independent Review of Children’s Social Care (2022), which called for a ‘whole system reset’ including an improved, multi-disciplinary ‘revolution in Family Help’ to improve outcomes for children and young people, and their families.

**Protocol registration:**

The full study plan is available here: https://youthendowmentfund.org.uk/wp-content/uploads/2024/07/REVIEWED-YEF-AC2-Feasibility-Pilot-Study-Plan-FINAL-July-2024.pdf and via the Open Science Framework: https://osf.io/s9bux/.

**Supplementary Information:**

The online version contains supplementary material available at 10.1186/s40814-025-01736-z.

## Background

Experience of violence or criminal exploitation outside the home (also known as extra-familial harm) can include child sexual and/or criminal exploitation; peer sexual abuse; child radicalisation; teenage abuse in intimate relationships; and serious violence in public places [1–8]. These harms can emerge in children and young people’s peer groups, public and school settings [2, 3], with adults outside of the family unit, within the wider community and/or online [8–11]. The impact of these harms on children and young people is wide-ranging and includes poor mental health, threats to physical health (including potentially fatal violence), criminalisation, and negative impacts on future outcomes and achievements [11]. Importantly, young people who have been coerced to carry out criminal activities can be treated as perpetrators rather than as victims. This can lead to potentially lifelong impacts, with this blurred victim/perpetrator role not easily responded to by services who may be better suited to working with either one or the other [8, 12, 13]. Furthermore, families may be impacted due to threats of violence and death to silence and control the victim or being forced to settle debts. This also results in victims being unable to speak openly to professionals who may be able to help [8].

Evidence suggests that children and young people at risk for extra-familial harm often reach ‘critical moments’ in their lives (such as being excluded from school, physically injured, or arrested), when a ‘decisive response’ is paramount in making a difference to their long-term outcomes [14]. In the United Kingdom (UK), there has been an increased focus amongst child protective services, policymakers, and researchers on extra-familial harms and how this can be supported by child protective systems, including a shift in UK welfare policy to include place-based approaches. This has led to the formation of local safeguarding partnerships between stakeholders such as crime reduction agencies and welfare systems [11]. Further, contextual safeguarding is a framework implemented across local authorities in England and Wales to necessitate that child protective systems: target the social conditions of abuse; incorporate extra-familial contexts in child protection legislative frameworks; use partnerships with individuals and organisations responsible for the spaces where young people spend their time; and measure contextual outcomes [15].

Recent research shows that multi-agency partnerships and child welfare agencies often do not prioritise the social conditions of abuse, but rather target individual behaviour [11]. This omission can negatively impact children and young people as it does not adequately address the contextual factors that increase risks of extra-familial harm. Firmin [12] suggests that the barriers to dealing with extra-familial harms are the policy and practice frameworks they are grounded in, not the legislation that deals with harms outside the home. For example, traditional practices amongst child protective and welfare services do not have a category of ‘extra-familial harms’ within their frameworks, resulting in them using tools that are designed for abuse or neglect.

The UK Government’s Independent Review of Children’s Social Care highlighted that the ‘*current children’s social care system was increasingly skewed to crisis intervention, with outcomes for children that continue to be unacceptably poor and costs that continue to rise*’, and that ‘*for these reasons, a radical reset is now unavoidable*’ [16]. Amongst its recommendations, the report called for changes to the children’s social care response so that children, young people, and families receive more responsive, respectful, and effective support. This included recommending the introduction of one multi-disciplinary Family Help Team that covers both early targeted help and child in need, to reduce referral and handovers between services/teams, and ensure the provision of meaningful support. Teams would be based in community settings that are known to and trusted by families (e.g. schools, family hubs), and composed of multi-agency professionals including family support workers, domestic abuse workers, mental health practitioners, and social workers. Critically, the service offered to children and young people, and families would be tailored to their needs, and that of the neighbourhood, identified via robust needs assessment and feedback from families.

However, to date, there is very little evidence on what an effective multi-agency approach to supporting children and young people, and families looks like, or the services they should provide, particularly in the context of extra-familial harm [17]. Given the emerging but limited evidence on such programmes, we present the protocol for a feasibility and pilot study of a specialist multi-agency team embedded in neighbourhoods to support children and young people, and their families, who are at risk of, or experiencing, violence or criminal exploitation outside the home (the Youth Endowment Fund [YEF] Agency Collaboration Fund: Supportive Home Programme [herein ACF2]; [18]). This study will explore dimensions of, and factors affecting implementation, early signs of change and change mechanisms, and the feasibility and options for progression to an impact study.

### Aims and research questions

This mixed-methods study will be implemented in two concurrent and complementary phases which aim to (i) assess the feasibility of programme implementation and (ii) assess the feasibility of, and pilot an impact evaluation, and present an options analysis/recommendation for an impact study. Research questions for each phase are presented in Table 1.

**Table 1:** Research questions

Feasibility of implementation	1a. Can the programme be implemented with fidelity to the programme/site-level theory of change and delivery framework? 1b. Does the programme implementation plan and/or theory of change need refining? 1c. What is the programme recruitment, retention, and reach across strands? 1d. Is there a clear and consistent set of eligibility criteria being adhered to across and activity strands, that also reaches keyworker pathway pilot targets? 1e. What does programme referral, engagement, support offer, and completion look like for children and young people (and their family/carers) through the key worker offer and other programme activities? 1f. What factors support or impede programme delivery (including consideration of intervention characteristics, referral pathways and information sharing; delivery and multi-agency partner capacity experience, partnership working, skills and attitudes; workforce composition; implementation support systems; and community/system level factors)? 1g. What are service users and practitioner’s views and experiences of the programme?
Feasibility of and pilot of an impact evaluation	2a. What is the level of consistency and standardisation of programme implementation across the five sites overall? 2b. Are sites aligned enough in their aims and approaches to make a collective impact study feasible? 2c. What is the feasibility of measuring impact at an individual, site, and programme level? 2d. What is the required sample size for a full impact evaluation? 2e. Is it feasible to achieve a sample size with enough power to progress to an impact study? 2f. Across sites, is the programme sufficiently distinct from business as usual for an impact evaluation to be feasible? 2g. What is the direction and magnitude of potential changes in identified outcomes, and does the programme achieve its intended outcomes? 2h. Are the piloted outcomes/measures appropriate/practical/reliable/valid for the programme? 2i. What are the options and considerations for design of an impact study (e.g. What potential is there for randomisation at individual or area level; do any sub-group effects need to be considered and why)? 2j. What scale of delivery would be required for the sample size to be met, given the evaluation design being recommended at the end of the feasibility study (e.g. how many sites, how many neighbourhoods in each site)? 2k. What research questions could a robust impact evaluation answer? 2l. What is the acceptability of an impact evaluation to programme stakeholders? 2m. Do sites have the capacity to scale up if the study progresses to a full impact evaluation (considering pilot recruitment/retention/reach and local needs/systems)?

### The intervention

**Fig. 1:**
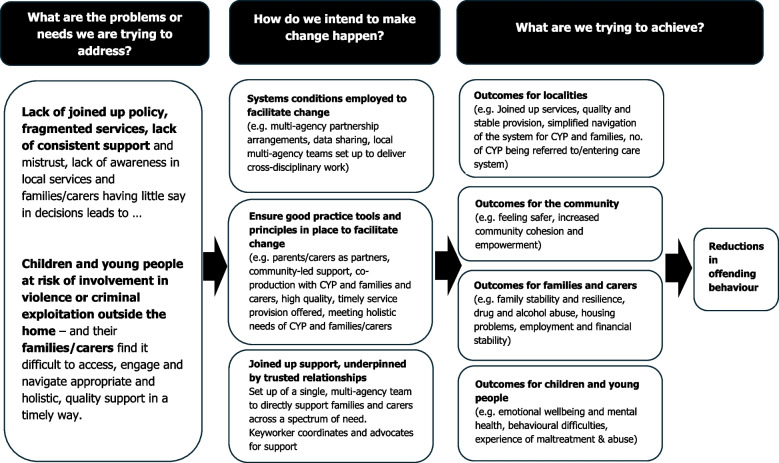
A priori high-level programme theory of change

The ACF2, commissioned and funded by YEF, will test specialist multi-agency and multi-disciplinary teams (referred to as multi-agency hereafter) located in neighbourhoods to support children and young people aged 10–20 years (and their families or carers) who are at risk of, or experiencing, violence or criminal exploitation outside the home [18]. Figure 1 presents the a priori high-level programme theory of change and Additional file 1 provides the detailed a priori programme theory of change (developed by YEF).

The programme will be delivered across five sites, and at site level the local programme will be led by the local authority. The multi-agency teams will consist of statutory organisations, voluntary, community, faith, and social enterprise (VCFSE) organisations, the police, probation, mental health professionals (for children and young adults), and education. The composition of each site’s multi-agency team will be based on local context, needs, and strengths and assets; thus, there will be some variation across sites (Additional file 2 presents a high-level summary of site-level programmes). Multi-agency teams will work within trusted community settings (e.g. community centres, libraries, schools), and key workers will build direct relationships with children and young people (and their families/carers where appropriate) to coordinate and advocate for support. The programme will combine work currently implemented at ‘targeted early help’, ‘child in need’, and ‘child protection’ and ‘in care’, and transitional safeguarding support for young adults aged 18–20 [16]. Support will be person/family-centred and strengths-based and thus the nature, type, and dosage of support will be determined by the individual needs of each child/young person/family unit. The targeted support for children and young people (and their families/carers) may be complemented by interventions delivered in the neighbourhood that aim to address the underlying causes of and contextual factors relating to extra-familial harm.

## Methods

### Setting and site selection processes

The programme will be delivered in ten neighbourhoods across five local authority areas in England and Wales (two neighbourhoods per site) to enable exploration of if and how different contexts influence implementation. These are Cardiff (East Cardiff neighbourhood [St Mellons, Llanrumney, and Trowbridge] and North Cardiff neighbourhood [Llanishen, Pentwyn, Pontprennau, and Llanedeyrn East]), East Sussex (Castle and Devonshire wards), Newham (East Ham and Plaistow), Swansea (East area and Penderry), and Swindon (one neighbourhood consisting of Park North, Park South, and Walcot East; and one neighbourhood consisting of Pinehurst and Penhill).

The independent evaluation team (led by authors ZQ/HS/FdV) and programme sites were selected using an application process commissioned by the YEF [18], who invited local authorities in England and Wales to apply for the Agency Collaboration Fund: A Supportive Home grant round. The funding aims to test specialist multi-agency and multi-disciplinary teams located in two neighbourhoods to support children and young people aged 10–20, and their families/carers who are vulnerable to—or experiencing—violence or criminal exploitation outside the home. The grant round funded up to five local authorities to put into action recommendations from the Independent Review of Children’s Social Care. Amongst other criteria (listed in full in [18]), eligible sites were those with high rates of youth violence and/or offending by children and young people across the local authority and within the selected neighbourhoods; potential for future programme scale up; and a good state of readiness to deliver the multi-agency programme. Applicants were shortlisted and invited to an interview panel that included YEF and members of the independent evaluation team. Five sites were selected from the shortlisted applicants based on evaluation criteria which included the extent to which the planned programme of work aligned with the YEF specification [18] and outcome framework [19]; evidence that the programme differs from ‘business-as-usual’; the feasibility of the site reaching 200 children and young people; alignment of neighbourhood sites with relevant geographies to enable area-level evaluation; and feasibility of availability and access to relevant local partnership data.

Successful applicants were supported through the preparation (~9 months) and implementation (11 months) phase with a YEF-appointed co-design partner. In the preparation phase, site-level partnerships prepared for programme implementation through the development of a local systems map; site-level theory of change, and partnership delivery model and associated policies. Sites progressed to programme implementation in April 2024.

### Sampling, participant recruitment, and eligibility criteria

#### Sample size

For the feasibility and pilot of the impact evaluation phase of this study, pre- and post-outcome measures will be collected from 1000 young people who receive key worker support (~200 per site). This sample size target was a pragmatic decision, agreed a priori between the evaluation team and the funder (YEF), on the basis that this would provide a good indication of intervention reach and of the ability of sites to recruit a sufficient number of participants to evaluate impact in a full trial. Our target number of 10 neighbourhoods exceeds the 8 identified by Hemming and colleagues in their overview of external pilot studies [20], and our total sample size exceeds the 30 participants per site recommended by Lancaster et al. [21] and the 35 per group recommended by Teare et al. for estimating the uncertain critical parameters (including standard deviation for continuous outcomes, and consent, event, and attrition rates for categorical outcomes) [22]. For the feasibility of the implementation phase of this study, interviews will be completed with 100–125 stakeholders (20–25 per site), 75 children and young people (15 per site), and 75 parents/carers (15 per site). Participants will be selected purposefully to ensure diversity across and within sites. For stakeholders, the number of participants will be guided by the delivery context of each multi-agency team and saturation. Children and young people and parents/carers will be purposefully sampled (in collaboration with gatekeepers who provide a safeguarding role) to ensure diversity in relation to socio-demographics and guided by saturation.

#### Recruitment and consent

As the delivery of the multi-agency programme will be closely embedded with the delivery of statutory services, in many cases, children and young people will have a legal right to receive the statutory elements of provision. Therefore, participants will consent to the programme and the evaluation as separate entities. Before children and young people can take part in the study, the correct consent procedure must be followed. Parent/carers of children aged 10–15 years may ‘opt-out’ their child (i.e. inform the key worker if they do not want the child to take part). For interviews with children aged 10–15 years, parent/carers have the opportunity to ‘opt-in’ their child (i.e. inform the key worker if they do want the child to take part). All children and young people will have the opportunity to assent/consent to their own participation.

#### Children and young people with key worker support (pre- and post-outcome measures)

Children and young people (and parents/carers as appropriate) will be provided with an introduction to the study and an invitation to take part via their key worker. This will include a verbal description of the study, provision of relevant (e.g. age and developmentally appropriate) information sheets, and an opportunity to ask questions and consider their participation. For children and young people, filling in the questionnaire implies their assent/consent. During this consent process, children and young people and their parents/carers must also consent for their data to be deposited in the YEF data archive.^1^ To be eligible for the study, children and young people need to be receiving key worker support, aged 10–20 years, at risk of or experiencing extra-familial harm, and have the capacity to provide informed consent/assent. Further, the key worker must deem that there are no current (or previous) safeguarding risks that would be impacted by the child or young person’s inclusion in the study that cannot be addressed through minor amendments to study design.

#### Parents/carers and children and young people (interviews)

Key workers will identify potential children and young people, and parent/carers to invite to participate in an interview. They will provide them with an information sheet and the participant will be able to ask their key worker any questions and will also be provided with contact details for the research team. The researcher will explain the study verbally again at the start of each interview; copies of participant information sheets will be available for participants, and they will have the opportunity to ask questions.

#### Stakeholders (interviews/focus groups)

Stakeholders will be purposefully selected to ensure diversity and capture the whole complex system surrounding each multi-agency team. Stakeholders will include delivery leads, multi-agency team members, and wider partners. Stakeholders must be over 18 years, able to give informed consent, and involved in the delivery of the multi-agency programme. Stakeholders will be approached by a gatekeeper (site-level programme lead) who will explain the study and ask if they are happy to have their contact details shared with the researchers. The researcher will provide them with a participant information sheet and give them the opportunity to ask questions before taking consent and arranging a suitable time and date for the interview. The study's purpose will be explained again verbally at the beginning of each interview and participants will be given the opportunity to ask questions.

### Data collection and outcomes

#### Assessment of routine programme monitoring data

We will work with delivery sites to set up routine monitoring/assessment processes to monitor all stages of delivery including programme uptake (e.g. number/profile of children and young people/families/carers referred/engaged/supported), dosage (e.g. number/type of interventions/referral pathways), distinction (from business as usual), and attrition, across activity strands. With participant consent, data will be linked via a pseudo-anonymised code to individual-level pre-post outcome measurements.

#### Review of programme documentation and refinement of programme description

We will collate and review programme documentation to add context to the study. This may include delivery plans (including theories of change), programme promotion material, steering group minutes, and YEF programme monitoring forms. The TIDieR-PHP (Template for Intervention Description and Replication for Population Health Programmes) reporting guideline will be completed with sites [23].

#### Qualitative data collection: interviews/focus groups

Interviews (virtual or via telephone^2^) will be undertaken with children and young people, parents/carers, and stakeholders to examine views and experiences of programme implementation and outcomes/impacts, co-production and feedback loops, and as relevant evaluation design and outcome measurements. General topic guide templates will be developed to ensure consistent coverage across participants, and adapted, as appropriate, for each participant group to reflect their varying roles within the programme. Questions will be age and developmentally appropriate and culturally sensitive.^3^ Public involvement and engagement (PPIE) consultations were carried out with young people engaged in youth services at site level to ensure the interview topic guide was acceptable and comprehensible to participants. Interviews will be semi-structured and approximately 30–60 min in duration. We will engage with practitioners at delivery months 3–4 (programme steering group, key workers, and members of the multi-agency team) and delivery months 10–11 (programme steering group, key workers, members of the multi-agency team, and wider community members) to enable timely feedback/adaptations prior to feasibility/pilot study completion. Engagement with children and young people, and parents/carers will be ongoing throughout the study period, with participants invited to interview at 12 weeks following formal programme engagement.^4^ If it becomes apparent that children and young people complete or drop out of the programme before the 12-week follow-up point, we will aim to engage with them at an earlier stage (e.g. 6 weeks).

#### Primary data (individual level)

Children and young people will be asked to complete a set of measures (described in Table 2) at baseline (point of engagement with a key worker) and follow-up (+12 weeks).^5^ We will collect demographic data including age, gender, ethnicity, and socio-economic status using questions from the England version of the World Health Organization Health Behaviour in School-aged Children [HBSC] cross-sectional survey [27]. Questionnaires will take no longer than 15 min to complete and baseline data will be collected alongside other routine service data to reduce the burden on children and young people. PPIE consultations were performed prior to delivery to ensure that measures were comprehensible and acceptable to participants. Facilitated by the key worker, questionnaires will be self-completed by children and young people while they are present at each delivery site using an online Qualtrics questionnaire via computer, tablet, or mobile phone depending on the IT infrastructure at each site. Sites may also have additional locally captured measures that are relevant to their programme which may inform the study. These will be identified at site level and shared as relevant to the data type (e.g. pseudo-anonymised routine monitoring data; anonymised case studies).

**Table 2:** Primary/secondary outcomes and associated measures for feasibility and pilot of impact evaluation

**Key outcomes**	**Measures**
**Primary outcomes**	
Availability and suitability of individual-, site-, and/or area-level routine data on violent offending	Routine crime data (e.g. police-recorded crime data; locally captured crime data)
Emotional regulation and behaviour of the children and young people (CYP)	Strength and Difficulties Questionnaire (SDQ) [24]
**Secondary outcomes**	
Mental health and wellbeing of the CYP	Mental health and wellbeing (Warwick-Edinburgh Mental Well-being scale [SWEMWBS]) [25]
Children and young people’s experience of the service	Child Experience of Care Questionnaire [CHI-ESQ] [26]
Availability and suitability of individual- and/or area-level data on victimisation	Victimisation data (e.g. from the Crime Survey of England and Wales [CSEW] and/or local site data)
Availability and suitability of individual- and/or area-level data on school exclusions	School exclusion data (e.g. from School Census, Ministry of Justice—Department for Education linked dataset)

#### Secondary data (individual, site, and programme level)

We will assess the availability of administrative data to measure outcomes (example of potential sources provided in Table 2) and assess the feasibility of using these to evaluate impact at the individual, site, and programme level. With stakeholders from each site, we will explore what data on crime, victimisation, and school exclusions are available, and if these can be made available and, for aggregate data, at what level of geographical (e.g. lower-super output area) and temporal resolution (e.g. weekly). We will similarly explore the availability of multi-agency data, Requirements for data sharing, including approval processes and requirements for secure storage, will be determined with each site/data provider. We will assess levels of missing data and censoring (e.g. crimes, school exclusions with fewer than five occurrences during the relevant time/geographical grouping, are often subject to censoring) and consider if/how this can be addressed. Requirements for data sharing, including required approvals and processes for secure storage, will be determined with each site/routine data provider.

### Data storage/management

Full details of data management and storage processes can be found in the full protocol, which is publicly available [28].

### Data analysis

#### Assessment of routine programme monitoring data

We will use descriptive statistics to describe programme delivery including programme uptake, dosage, and attrition. Data will be linked to individual-level outcome measurements via a pseudo-anonymised code.

#### Interviews

With participants’ permission, interviews will be audio recorded (using MS Teams or a voice recorder) and transcribed verbatim (and checked for accuracy) for analysis and anonymised. For those who do not consent to be recorded, interviewers will take handwritten notes during and immediately following the interview. We have used Normalisation Process Theory (NPT) to develop interview schedules and to provide a structure for the presentation of qualitative analysis [29]. NPT describes important individual and organisational factors that are likely to have influenced the embedding of the programme into practice, including how multiple stakeholders made sense of the multi-agency approach (coherence); their willingness to commit to the work required (cognitive participation); their ability to take on the work required (collective action); and activity undertaken to monitor and review implementation independently of the evaluation (reflexive monitoring). This approach will also allow us to capture important qualitative information on the multi-agency approach including (i) acceptability; (ii) unforeseen resource/capacity implications; (iii) contextual factors influencing engagement; (iv) perceived mechanisms of effect; (v) perceived unintended consequences; and (vi) stakeholders’ experience of co-production with children, young people, and families. Data will be analysed using deductive (based on NPT themes) and inductive (based on emerging themes) approaches. Verbatim interview quotations will be provided to support key findings.

#### Review of programme documentation

Programme documentation will be reviewed and summarised to contextualise findings. Outputs will be reviewed with consideration of the deductive and inductive themes derived through analyses of interviews. Examples of programme documentation may be used to provide support for key findings.

#### Assessment of primary and secondary data

As this is a feasibility and pilot study, no inferential analyses will be undertaken. Descriptive analyses of all key outcomes will be conducted to provide an overview of data, to describe the target group and properties of aggregate datasets.

#### Progression to full impact evaluation criteria

Criteria for progression to a full impact evaluation study are presented in Table 3. We will review and discuss the extent to which findings from the feasibility and pilot study support progression to a full impact study, based on the criteria outlined in Table 3 with YEF (the funders; 18).

**Table 3:** Criteria for progression to a full impact evaluation study

**Criteria**	**Indicator**	**Criteria to inform progression to full impact evaluation**		
		**Fully met**	**Partially met**	**Not met**
**Feasibility of implementation (phase 1)**				
** Creation of programme-level theory of change**	Agreed by YEF, LJMU, RIP^a^	Yes		No
** Creation of site-level theory of change**	Agreed by YEF, LJMU, RIP	Yes		No
** Creation of site-level system map**	Agreed by YEF, LJMU, RIP	Yes		No
** Ability of programme to be implemented as planned (fidelity)**	Agreed by YEF, LJMU, RIP, and delivery leads	Yes	Yes, with relevant adaptations	No
** Ability of programme to receive appropriate referrals**	Proportion of participants that meet programme inclusion criteria	70–100%	40–69%	0–39%
** Ability of programme to engage participants**	Proportion of participants consenting to intervention	70–100%	40–69%	0–39%
** Ability of programme to retain participants**	Proportion of participants who attend programme intervention activities	80–100%	40–79%	0–39%
** Ability to collect routine monitoring data**	Proportion of missing baseline data on programme participants captured by data systems	0–35%	36–50%	51–100%
**Feasibility and pilot of impact evaluation (phase 2)**				
** Ability to collect CYP baseline measures**	Proportion of CYP completing baseline questionnaires	60–100%	40–59%	0–39%
** Ability to collect CYP follow-up measures**	Proportion of CYP completing follow-up (+3 months) questionnaires	60–100%	40–59%	0–39%
** Outcome measure data completeness**	Proportion of missing data for each primary and secondary outcome measure	0–39%	40–59%	60–100%
** Availability of routine data for site-specific selected important outcomes**	Agreed by YEF, LJMU, and UoB. Proportion of outcomes for which data can be made available	60–100%	40–59%	0–39%
** Linked individual-level outcome data from routine sources can be made available and, if not, area-level routine outcome data can be made available at a sufficiently disaggregated level**	Agreed by YEF, LJMU, and UoB. Data can be made available at the individual level, or appropriate area level aggregation (appropriate geographical area to be determined in collaboration with sites, based on target area of intervention and hypothesised geographical reach of associated impacts)	Yes		No
** Outcome data can also be made available for small numbers without high levels of censoring**	Uncensored, anonymised, small area level data	≤20% of primary outcome data censored	>20% of primary outcome data censored; appropriate imputation methods can be applied	>20% of primary outcome data censored; appropriate imputation methods cannot be applied
** Outcome data are available at an appropriate level of temporal aggregation**	Primary outcome data available at monthly intervals or less	Primary data available at ≤ monthly intervals^b^	Primary data available at quarterly intervals	Primary data are available at intervals of more than a quarter^c^
** Outcome data are available for control sites**	Primary outcome data available at monthly intervals or less	Primary data available ≤ monthly intervals	Primary data available at quarterly intervals	Primary data are available at intervals of more than a quarter
**Additional criteria**				
** (Adverse) effects of programme participation**	From preliminary analysis of outcome data and qualitative data	No evidence of substantial negative effects of participation		Evidence of substantial negative effects of participation
** Acceptability of programme activities**	From analysis of qualitative data	Target groups report programme activities and interventions are acceptable and/or could be feasibly improved	Target groups report programme activities/interventions are unacceptable and cannot identify how they could be improved. Sites develop plan to increase acceptability	Target groups report programme activities/interventions are unacceptable and cannot identify how they could be improved. Sites cannot identify a plan to increase acceptability
** Acceptability of evaluation methods**	From analysis of qualitative data	Target groups report evaluation methods are acceptable and/or could be feasibly improved	Target groups report evaluation methods are unacceptable and cannot identify how they could be improved. Evaluator/sites develop a plan to increase acceptability	Target groups report evaluation methods are unacceptable and cannot identify how they could be improved. Evaluator/sites cannot identify a plan to increase acceptability
** Programme implementation**	From analysis of theories of change and system maps, and interviews with providers	Programme is coherent: meets criteria for multi-agency approach and is distinct from business as usual	Programme is not coherent: does not meet criteria for multi-agency approach and is not distinct from business as usual. Sites identify plan to improve coherence	Programme is not coherent: does not meet criteria for multi-agency approach and is not distinct from business as usual. Sites cannot develop a plan to improve coherence

^a^RIP—Research in Practice, the programme co-design partner

^b^E.g. weekly

^c^E.g. 6-monthly, annually

#### Impact evaluation design/options appraisal

Initial consultation with YEF and delivery sites suggests that it may not be feasible to conduct a randomised controlled trial (RCT) to evaluate the programme [30]. However, as this would give the strongest evidence on whether the programme is effective, we will explicitly explore the possibility of evaluating the programme using this design with sites. For administrative data, as only two neighbourhoods will be purposively selected within each study site, randomisation at neighbourhood level within local authorities will likely not be possible. We will therefore particularly focus on the selection of control areas. If a RCT is not feasible, we will explore a quasi-experimental (natural experiment) evaluation design, using the ‘Target Trial Framework’ to make explicit where a future evaluation will mimic, and where it deviates, from the ideal target trial (RCT) [31]. Importantly, this will include the selection of optimal potential control areas for matching [32]. We will explicitly develop mitigation evaluation design elements for those domains where the quasi-experimental evaluation design will have to deviate from the ideal target trial, to optimise the strength for causal conclusions for the outcomes of interest. Once the optimal design and data elements for the site/programme-level administrative data evaluation have been determined, guided by the developed site-specific TTF matrices, we will explore issues around the use of multiple controls and consideration of various quasi-experimental techniques (e.g. propensity score matching, interrupted timeseries) to improve causal inferences through triangulation. Subsequently, we aim to synthesise the various qualitative and quantitative findings to outline the causal pathways and system in which these are embedded, using a ‘Research Synthesis by Configuration’ approach to describe where findings agree, contradict, extend, explain, or otherwise modify each other, to form a coherent narrative [33].

## Discussion

This feasibility and pilot study will provide an important and timely contribution to the emerging, but limited, evidence base surrounding the implementation of place-based multi-agency interventions to support children, young people, and their families at risk of extra-familial harm [34]. This work has direct implications for informing policy and practice in the wake of the Independent Review of Children’s Social Care (2022 [16]), which called for a ‘whole system reset’ including an improved, multi-disciplinary ‘revolution in Family Help’ to improve outcomes for children and young people, and their families. Based on the findings of this study, we will develop and assess the feasibility of progression to a full impact evaluation, which would provide a significant contribution to the evidence base [30].

Strengths of this study’s approach include rigorous co-development of programme-level and site-specific theories of change and local systems map to further support moves towards more holistic context-driven conceptualisations of the factors influencing extra-familial harms, and multi-agency approaches to address these factors using a place-based approach [11, 17, 30]. Our approach is further strengthened by tailored, diverse, and inclusive approaches to stakeholder engagement and data collection spanning the individual, site, and programme level. The use of the Target Trial Framework [31] and careful consideration of appropriate controls, and methodological triangulation will also ensure that any future full impact evaluation will be as robust as possible, maximising the strength of causal inferences.

The study has some limitations. Ideally follow-up data would be collected at +6 months, as behavioural change and changes to wider family/contextual factors are expected to take time. Further, despite the inclusion of areas with high levels of youth violence and/or exploitation, the incidence of engagement in violence or criminal exploitation during this time period may be low. This may lead to difficulties in detecting any early markers of change. However, due to the piloting period being 11 months, and the uncertainty of when and how well recruitment will proceed, and if so, how long children and young people will stay engaged in the programme, for the pilot period we are implementing a +3-month follow-up period to ensure that baseline and follow-up data are collected. This will mean that the follow-up data collection period may not mimic a future impact study design. We also note that while our criteria for progression to a full impact evaluation study apply to both treatment and control groups, for pragmatic reasons data collection and outcome scoping have been limited to participating sites (treatment groups) within this feasibility and pilot study. Considerations around the availability and suitability of outcome data for control sites will be considered as part of control site selection if this study proceeds to full impact evaluation.

A key output from this study will be a feasibility and pilot study report. This will include an updated programme-level theory of change and clearly defined programme implementation model(s); key lessons from programme implementation and recommendations for refinement; and an assessment of the feasibility of progressing to an impact study including considerations and options for progression to an impact study using the Target Trial Framework [31]. This will not only build on the limited evidence base for these types of programmes, but also support the development of robust approaches for evaluating complex place-based approaches for preventing violence amongst children and young people [34]. Involving the community in place-based evaluations is critical [30]. For this study, children and young people have informed study design and implementation (i.e. review of research tools and approaches) and will be key to developing a child-friendly summary of the feasibility and pilot study, ensuring findings are disseminated to those the programme aims to support.

## Supplementary Information

Additional file 1: Detailed a priori programme-level theory of change.

Additional file 2: High-level summary of sites and programme activities.

## Data Availability

The datasets generated and/or analysed during the current study will be deposited in the YEF data archive. This is a standard approach for all YEF-funded interventions and evaluations and has standard practices, information sheets, and consent forms which are used across all YEF evaluation partners (including academic institutions). The full protocol for depositing data to the YEF data archive is available here: https://youthendowmentfund.org.uk/wp-content/uploads/2023/11/YEF_Data_Protection_Evaluators-Oct-2023.pdf. The full study protocol plan is available here: https://youthendowmentfund.org.uk/wp-content/uploads/2024/07/REVIEWED-YEF-AC2-Feasibility-Pilot-Study-Plan-FINAL-July-2024.pdf. Links to any relevant publicly available data sources will be provided in our outputs for this pilot and feasibility study.

## Abbreviations

ACF2: Agency Collaboration Fund: Supportive Home Programme
CYP: Children and young people
HBSC: Health Behaviour in School aged Children
LJMU: Liverpool John Moore’s University
NPT: Normalisation Process Theory
PPIE: Public involvement and engagement
RCT: Randomised controlled trial
TIDieR-PHP: Template for Intervention Description and Replication for Population Health Programmes
TTP: Target Trial Framework
UK: United Kingdom
VCFSE: Voluntary, community, faith, and social enterprise
YEF: Youth Endowment Fund

## Footnotes

1 This is a standard approach for all YEF-funded interventions and evaluations and has standard practices, information sheets, and consent forms which are used across all YEF evaluation partners (including academic institutions). The full protocol for depositing data to the YEF data archive is available here: https://youthendowmentfund.org.uk/wp-content/uploads/2023/11/YEF_Data_Protection_Evaluators-Oct-2023.pdf.

2 If deemed necessary to improve engagement with children and young people, processes will be established with sites to implement in-person interviews.

3 Participant information sheets, interview schedules, and questionnaires have been piloted with children and young people across study sites, to ensure comprehension, feasibility, and that completion is possible in the allocated time.

4 I.e. key worker has conducted needs assessment and child/young person and/or parent/carer has agreed to participate in the programme and evaluation.

5 With the exception of the Child Experience of Care Questionnaire [CHI-ESQ], which will be completed at +3 months only.
