# Supplementary figures and images for: Protocol for a feasibility and pilot study of the implementation and impact of specialist multi-agency teams supporting children and young people at risk of, or experiencing, violence or criminal exploitation outside the home
Source: Pilot Feasibility Stud. 2025 Nov 25;11:148. doi: 10.1186/s40814-025-01736-z (PMC12649095; doi:10.1186/s40814-025-01736-z)

**Additional file 1: Detailed a priori programme level theory of change**


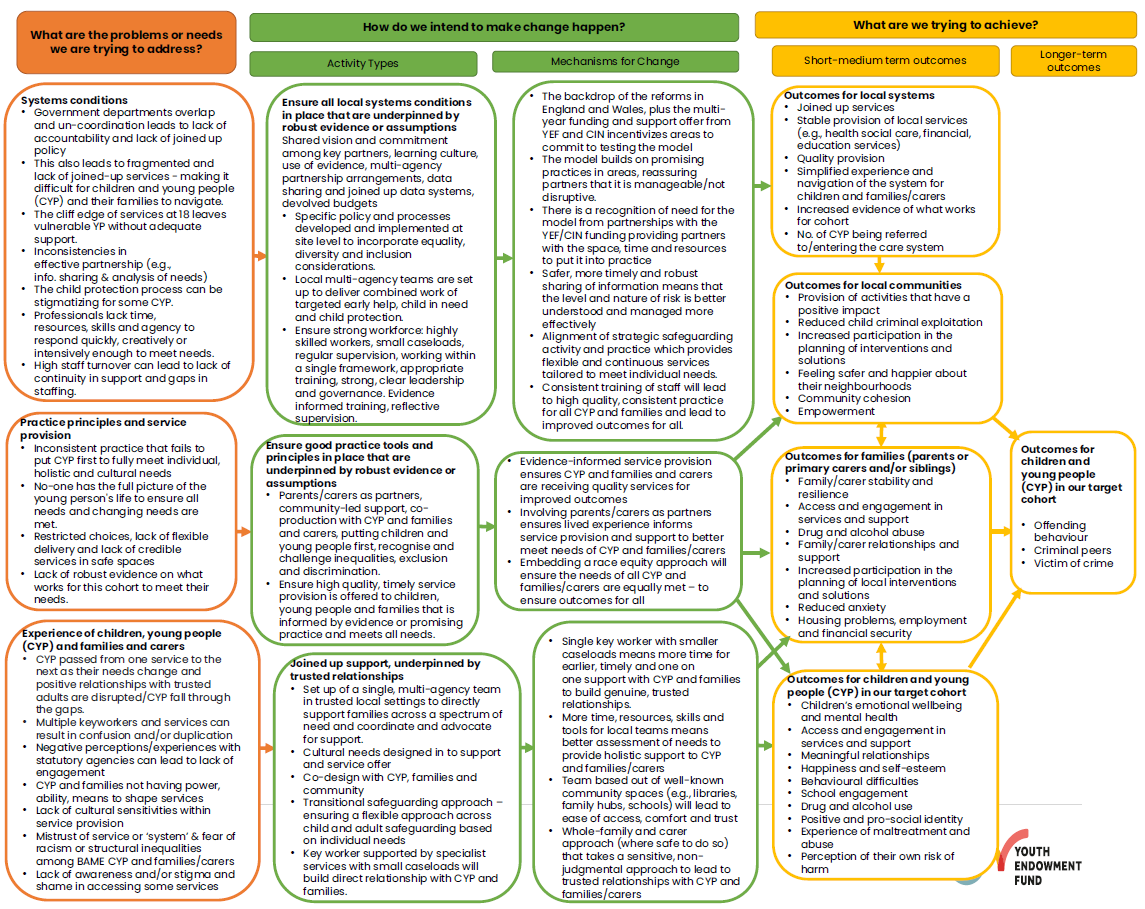

Supplement: Supplementary file 1 — Additional file 1: Detailed a priori programme-level theory of change. [file 40814_2025_1736_MOESM1_ESM.docx]
