# Supplementary material for: Protocol for a feasibility and pilot study of the implementation and impact of specialist multi-agency teams supporting children and young people at risk of, or experiencing, violence or criminal exploitation outside the home
Source: Pilot Feasibility Stud. 2025 Nov 25;11:148. doi: 10.1186/s40814-025-01736-z (PMC12649095; doi:10.1186/s40814-025-01736-z)
Supplement: Supplementary file 2 — Additional file 2: High-level summary of sites and programme activities. [file 40814_2025_1736_MOESM2_ESM.docx]

**Additional file 2: High-level summary of sites and programme activities**

| **Site** | Cardiff: Keeping and Staying SAFE YEF Project | East Sussex | Newham: Thriving Communities initiative | Swansea: YEF Safer Homes | Swindon |
| --- | --- | --- | --- | --- | --- |
|  | East (St Mellons, Llanrumney & Trowbridge)/North (Llanishen, Pentwyn, Ponprennau & Llandederyn East) | Castle and Devonshire wards | East Ham & Plaistow | East area and Penderry | Park North Park South and Walcot East; Inehurst and Penhill |
| **Programme activities** | **1. Children and young people (CYP) key worker offer:** assessment by key worker (lead practitioner) and support/mentoring from key worker and/or multi-agency team based on the needs of the CYP. Support offer co-produced with CYP and may include activities/sessions/1-2-1 work, and links to wider offer of support (e.g. early intervention offers for CYP). ***Eligibility criteria:*** CYP age 10-20 years at risk/experiencing youth violence or criminal exploitation. The offer and eligibility criteria vary by site, based on local needs and partnership arrangements. ***Sample size:*** Minimum 100 per neighbourhood. | | | | |
|  | **2. Multi-agency support offers for parents/carers/family:** The offer and eligibility criteria vary by site based on local needs and partnership arrangements. The multi-agency support will be offered to parents/carers of CYP who are engaged in the key worker offer and may also be offered to other parents/carers in the wider community. Activities may include: 1-2-1 work; family conferencing; peer support groups/networks; parent as partners forums; emotional wellbeing support; and group programmes (e.g. non-violence resistance; CACE). | | | | |
|  | **3. Early intervention offers for CYP/peer groups:** The offer and eligibility criteria vary by site, based on local needs and partnership arrangements. Activities may include school-based education programmes/workshops on topics such as social skills development; substance use; youth violence and exploitation; peer group assessments and support for CYP at risk of youth violence/exploitation; CYP forums to discuss youth violence and exploitation and inform prevention approaches; and positive and diversionary activities/outreach work. Activities may be co-produced with CYP. CYP who received the key worker offer may be referred into these activities, and CYP engaging in these activities may be referred into the key worker offer. | | | | |
|  | **4. Contextual safeguarding and community safety approaches:** These are dependent on local needs and existing partnership arrangements. Activities include multi-agency contextual safeguarding assessments, responses and interventions focused on specific locations and/or peer groups; multi-agency community safety planning; workshops to engage communities in keeping people safe, including activity co-production; and school based assessments to identify needs and inform interventions (e.g. staff training in trauma-informed approaches); and inter-generational workshops to encourage shared views of what works to keep communities safer. | | | | |
|  | **5. System wide activities:** These are dependent on local needs and existing partnership arrangements. Examples of system-wide activities include changes to partner assessment procedures to identify CYP at risk/experiencing youth violence or criminal exploitation; training for practitioners (e.g. mentoring approaches) or knowledge exchange activities (e.g. to help professionals understand how peer networks can impact safety and what interventions can be put in place); and CYP and parent/carer forums to discuss youth violence and exploitation and inform prevention approaches (linked to activity 2/3); and inter-generational workshops to encourage shared views of what works to keep communities safer (linked to activity 4). | | | | |
